# Vigorously cited: a bibliometric analysis of the 100 most cited sedentary behaviour articles

**DOI:** 10.1186/s44167-023-00022-8

**Published:** 2023-07-01

**Authors:** Aamir R. Memon, Sitong Chen, Quyen G. To, Corneel Vandelanotte

**Affiliations:** 1 Institute for Health and Sport, Victoria University, Melbourne, Australia; 2 Appleton Institute, Central Queensland University, Rockhampton, QLD Australia

**Keywords:** Activity behaviour, Lifestyle behaviour, Publications, Sedentary time, Sitting time

## Abstract

**Objectives:**

No citation analysis has examined peer-reviewed sedentary behaviour research articles, which is needed to assess the impact of this research and identify knowledge gaps. Therefore, this study aimed to identify the 100 most cited sedentary behaviour articles and examine their bibliometric characteristics.

**Methods:**

All databases indexed in the Web of Science database were searched in October 2022, and bibliometric characteristics of the studies, irrespective of the publication year, were imported and calculated. Descriptive statistics and visualisations by the VOSviewer were used for the presentation of bibliometric characteristics.

**Results:**

The 100 most cited articles received 49,062 citations in total, with a median citation density of 32.5 citations per article per year. The majority of included articles were reviews (n = 48; 22,856 citations), focused on adults (58%; 26,809 citations) and reported on the relationship of sedentary behaviour with health (n = 64; 34,598 citations); more specifically they focused on anthropometric indices (28%), metabolic health (24%), and mortality (23%). The United States was ranked first in terms of the overall for most cited articles. However, Australia was ranked first for institutions and authors contributing to the most cited sedentary behaviour articles.

**Conclusions:**

Papers published after 2007 were predominant in the list of 100 most cited sedentary behaviour papers, as were those focusing on associations with physical health outcomes and those focusing on adults. While original articles were cited more, discussion papers had more impact on the field as they received more citations in less time. Research examining associations between sedentary behaviour and health was cited more. The field is dominated by contributions from high-income countries.

**Supplementary Information:**

The online version contains supplementary material available at 10.1186/s44167-023-00022-8.

## Introduction

Several systematic, scoping, and umbrella reviews have concluded that sedentary behaviour is negatively associated with important health outcomes, including cardiometabolic risks, mortality, certain types of cancers, and changes in body composition [1–11]. Breaking up or reducing prolonged sitting, on the other hand, is associated with various health benefits [12–14]. Furthermore, there are now public health guidelines specifically suggesting limiting prolonged sedentary time in all age groups [15, 16]. Therefore, policy and interventions for reducing and breaking up sitting time are essential for better health outcomes.

From a historical perspective, first study on sedentary behaviour was conducted by Morris and colleagues, which examined cardiovascular events in sedentary bus drivers in London [17]. However, sedentary behaviour and physical inactivity were not recognised as two distinct health behaviours for several decades after the Morris et al. study [17]. That is, the term ‘sedentary’ was used to indicate inability to meet physical activity recommendations [18, 19]. It was not until the 1990s that a small number of public health researchers begun to create a paradigm shift which enables recognition of physical inactivity and sedentary behaviour as two health behaviours independently affecting health [19–24]. It was only in 2012 when the Sedentary Behavior Research Network [25, 26] and, later on, some time-use epidemiologists [27, 28] classified sedentary behaviour as “*a distinct yet co-dependent behaviour competing for time with physical activity and sleep throughout the day*” [1]. Since then there has been a rapid rise in sedentary behaviour research [1]. While identifying the most seminal sedentary behaviour articles with a long-lasting impact in the field can be difficult, it can be beneficial in several ways.

Bibliometric analyses are a useful way to understand research focus and publication output in a specific field, which can help to determine research trends [29, 30]. Citation analysis, a type of bibliometric analysis, determines most cited papers in a field by quantifying them according to the citation count [31–35]. In addition, by identifying the most seminal papers in the field (i.e., what research is cited the most, where is the focus of the field), citation analysis can be useful in finding knowledge gaps, which may eventually help move the field forward [31, 35]. Furthermore, the list of most cited papers in a field may be used as a guiding tool for students and new researchers [32, 34]. Several methods have been used to determine the number of most cited papers in a citation analysis depending on the breadth of the field of research [36–38]. For example, some studies present either the top 1% most cited papers [37], only top 50 most cited papers [31–33], or papers with at least 400 citations [34, 38].

Recently, a citation analysis of 500 most cited physical activity papers was conducted [39]. However, as physical inactivity and sedentary behaviour are now recognized as two distinct health behaviours, there is merit in examining the bibliometric parameters of the most cited sedentary behaviour papers as well. To the best of our knowledge, no study to date has focused on identifying the most cited publications in sedentary behaviour research. Therefore, this study aimed to identify 100 most cited papers in sedentary behaviour and present their bibliometric characteristics.

## Methods

### Study design

The methodology used in this study was informed by a previous bibliometric study on top-cited physical activity papers [39]. The relevant literature for this bibliometric study (i.e., citation analysis) was searched using the “all databases” option in the Web of Science database (Clarivate Analytics, USA) as it enabled us to cover the papers indexed in the MEDLINE database. The Web of Science database was preferred over Scopus database for the current study because the citation data in Scopus database only covers articles published after 1996 [40]. As this study did not involve human participants or animal models, ethical approval was not required.

### Study selection and search strategy

To be included, peer-reviewed journal publications focusing on sedentary behaviour as the main topic from a behavioural and public health point of view (including guidelines, policy statements, discussion papers, validation of sedentary behaviour assessment methods, and interventions aiming to reduce or interrupt sedentary behaviour) were considered, without any restriction for study design (e.g., observational, experimental), type of publication (e.g., editorial, brief report, reviews), language of publication (e.g., English, Spanish), or year of publication. The main focus of this study was to examine sedentary behaviour publications from a broad behavioural and public health view, not from a physiological point of view. We chose to include the 100 most cited papers in this study bibliometric study as it ensures that a wide variety of paper types will be included (i.e., not just systematic reviews, but also original research studies, policy papers/guidelines) [39].

The exclusion criteria were: (1) papers focusing on physical inactivity or sleep (e.g., “*calibration of two objective measures of physical activity for children*” or “*short sleep duration is associated with increased obesity markers in European adolescents: effect of physical activity and dietary habits. The HELENA study*”); (2) papers focusing on exercise physiology (e.g., “*heart rate variability and autonomic activity at rest and during exercise in various physiological conditions*” or “*hemodynamic response to work with different muscle groups, sitting and supine*”), biomechanics/ergonomics (e.g., “*are neck flexion, neck rotation, and sitting at work risk factors for neck pain? results of a prospective cohort study*” or “*low back joint loading and kinematics during standing and unsupported sitting*” or “*sitting comfort and discomfort and the relationships with objective measures*” or “*the effect of different standing and sitting postures on trunk muscle activity in a pain-free population*”), and/or physiotherapy or rehabilitation (e.g., “*accelerometers in rehabilitation medicine for older adults*” or “*effect of neck exercise on sitting posture in patients with chronic neck pain*”); and (3) studies conducted on animals (e.g., “*inducible depletion of satellite cells in adult, sedentary mice impairs muscle regenerative capacity without affecting sarcopenia*”) and/or in controlled condition or laboratory studies (e.g., “*comparison of pedometer and accelerometer accuracy under controlled conditions*”). Furthermore, the focus of each included publication had to be solely on sedentary behaviour (studies that examined physical activity as a co-variate or mediator of sedentary behaviour were still eligible to be included if the overall focus of the paper was still on sedentary behaviour). As such, papers that focused on physical activity and sedentary behaviour in combination were excluded (e.g., “*physical activity and sedentary behavior in people with major depressive disorder: a systematic review and meta-analysis*” or “*physical activity and sedentary behavior among schoolchildren: a 34-country comparison*”). Sedentary behaviour was defined as “*any waking behaviour characterized by an energy expenditure* ≤ *1.5 metabolic equivalents (METs), while in a sitting, reclining or lying posture*” [26]. Therefore, papers reporting on “physical inactivity” – insufficient level to meet physical activity recommendations – to denote sedentary behaviour were also excluded.

The search strategy was developed based on a previous review [39], and the initial search strategy was pilot tested and assessed by all the authors to check for any modifications. This was done several times to finalise a search syntax that covers all relevant papers, with minimal false positives. The literature search was conducted on October 21, 2022, and papers with the following keywords in the title and/or abstract and/or keywords plus were searched: sedentariness, sedentary behaviour, sitting, reclining time, stationary behaviour/time, sedentary time, non-screen-based sedentary time, television (TV) watching or viewing, video watching, internet use, gaming/video games or electronic game playing, social/electronic media, screen time, small screen, media time/use, smartphone/mobile phone/cell phone use, app use, and PC/computer/tablet use or time. The detailed search strategy was added as Additional file 1.

### Identification and assessment of papers

Initially, as a test, 50 randomly chosen highly cited papers on sedentary behaviour were reviewed by all the authors to refine the eligibility criteria. Then, a list with the 500 most cited papers was reviewed by all the authors. Finally, a list of 2,000 most cited papers was independently assessed by two authors (ARM, SC). This number of papers was to ensure enough eligible papers would remain when all those not eligible were removed. Any disagreements were resolved through consensus or by involving a senior author (CV).

**Fig. 1:**
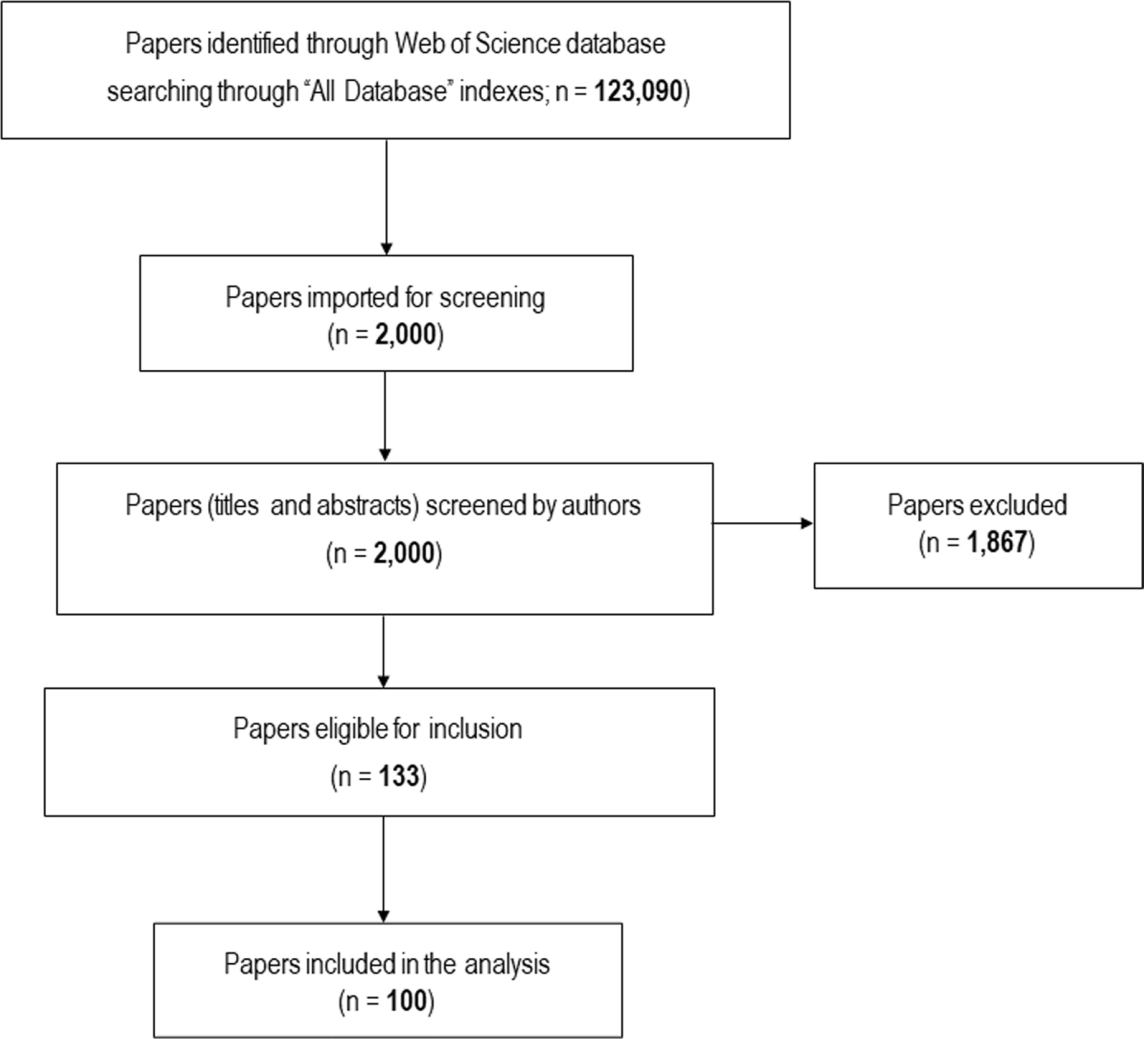
Identification and assessment of articles

The eligibility of papers was determined through screening the titles and abstracts of the papers by all authors. When the title and abstract did not provide sufficient information, the full-text of such papers was assessed in a consensus meeting. The process of literature search, screening, and inclusion of papers in the current study is presented in Fig. 1. Only the highest cited version of duplicate papers was included, their lower cited version was included in the list but not ranked.

The final list of 100 most cited papers was compiled from the information that was extracted from the Web of Science database, including the number of citations, publication year, citation density, publishing journal, and author’s country and institution of affiliation. The bibliometric parameters imported from the Web of Science database were based on the affiliation details mentioned in the paper at the time of its publication. If an author had more than one affiliation listed on the paper, then the data of those all affiliations were imported. The country of affiliation of authors was used to determine single-country (national) and multi-country (international) collaboration. Citation density was determined by dividing the number of citations by the number of years since publication of the paper. We extracted journal-based metrics up to the year 2021, including journal impact factor and journal citation indicator from the Web of Science database. Information on publishing model was obtained from the Web of Science database.

On the basis of number of authors, articles were categorised as single-authored, double-authored, and multi-authored publications. Information on different age groups covered within the articles was obtained from their full-texts and categorised as: children and adolescents, adults, and combined age groups. Information on health outcomes overed within the articles was also obtained from their full-texts and categorised as: mortality (e.g., all-cause, cardiovascular, cancer mortality or mortality from other causes), cardiovascular disease, diabetes, cancer, metabolic health (e.g., blood pressure, glucose, lipids), anthropometric indices (e.g., body mass index, waist circumference, skin fold), fitness (e.g., cardiorespiratory fitness), psychological health (e.g., self-esteem, pro-social behaviour, anxiety, and depression), cognition (e.g., memory, academic performance), bone health, sleep, pain, asthma, sciatica, musculoskeletal problems (e.g., low back pain), and fatigue.

Based on a previous bibliometric study [39], we classified the papers into original articles, reviews and policy papers/practice guidelines. However, we added an additional category (i.e., discussion papers) for papers that did not fit in those categories. Original articles were further classified into: (a) validation or evaluation papers, (b) observational (e.g., cross-sectional, cohort, case–control) or qualitative studies, and (c) interventional (e.g., randomized, non-randomized) studies. The complete list of 100 most cited papers stratified by the type of paper is presented as Additional file 2. Furthermore, we classified the papers into six groups in accordance with the Behavioural Epidemiology Framework: (1) Relationship of sedentary behaviour with health outcomes; (2) Measurement and assessment of sedentary behaviour; (3) Prevalence and epidemiology of sedentary behaviour; (4) Determinants and correlates of sedentary behaviour; (5) Interventions and programs to reduce and/or interrupt sedentary behaviour; and (6) Public health guidelines and policy for sedentary behaviour. [1, 41–43].

### Statistical analysis

We used SPSS v26 for data analysis, and findings were presented as counts (or percentages), minimum–maximum, mean or median, and standard deviation. Spearman’s correlation coefficient was used to determine the association between the number of citations and the number of years since publication, with a p-value of < 0.05 considered statistically significant. Co-authorship and keyword analysis was performed using the VOSviewer software (Leiden University, Netherlands) to examine collaborative networks between authors and most commonly used keywords in most cited sedentary behaviour papers [44].

## Results

### Publication and citation characteristics

**Fig. 2:**
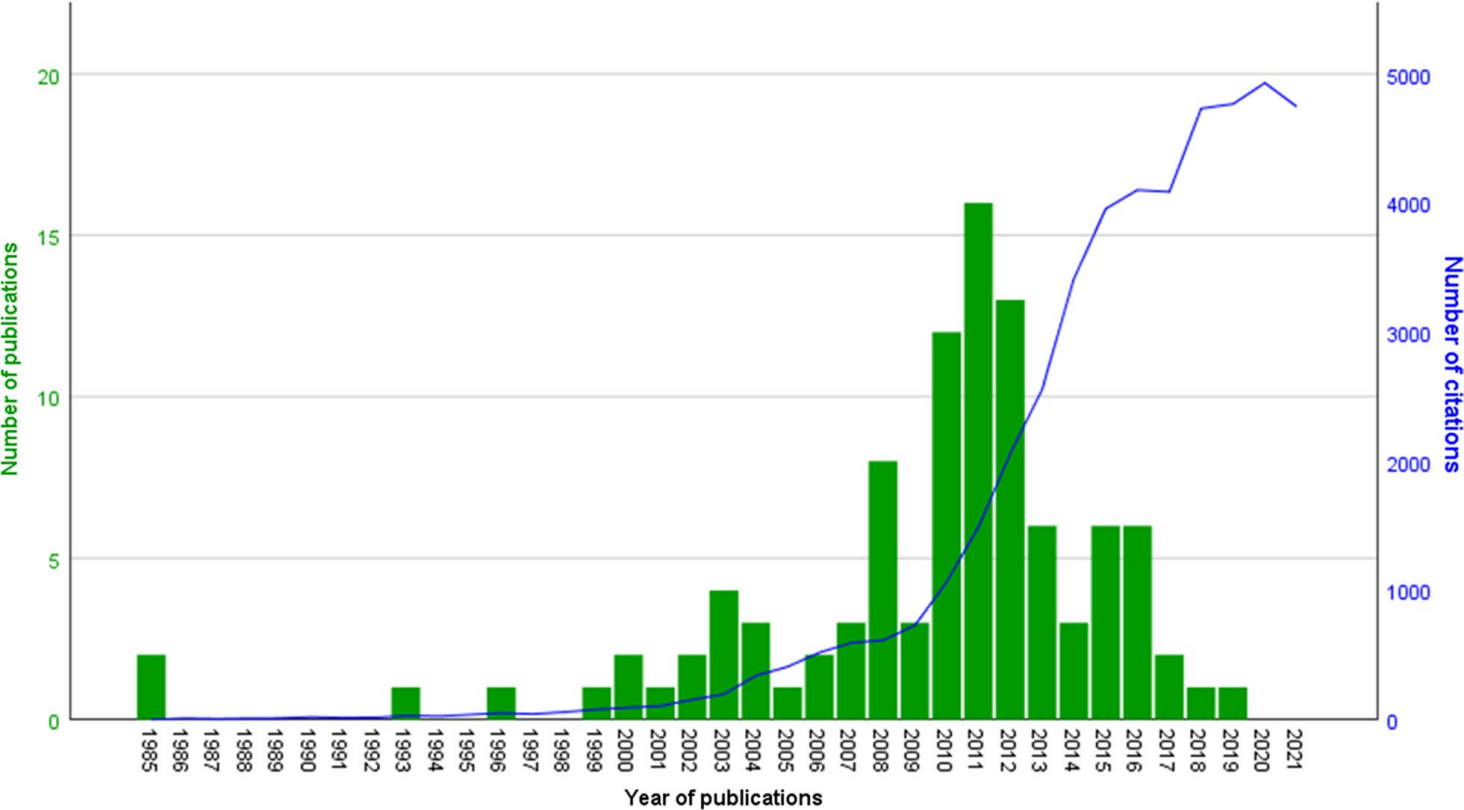
Yearly distribution of publications and citations for 100 highly cited papers

A total of the top 100 papers were included in this study which were published between 1985 and 2019. The highest number of most cited papers (n = 31) was recorded during 2010–2012. In total, the 100 most cited papers received 49,062 citations, with a citation density of 4672.8 citations per year. The average number of citations was 490.6 ± 361 (median = 335), with a range from 202 to 1779 (Table 1). The median citation density was 32.5 citations per article per year, ranging from 6.1 to 262 (mean = 46.7 and SD = 44). The number of citations continuously grew over time after 1997, whereas a sharp increase was observed for papers published after 2009.The distribution of the number of papers and citations by year is shown in Fig. 2. Three articles were single-authored, 9 were double-authored, and 88 were multi-authored publications. In terms of study population, 25% articles focused on children and adolescents, 58% on adults, whereas 12% combined age groups, and 5% were unclassified. The health outcomes covered in top-cited sedentary behaviour articles included mortality (23%), cardiovascular diseases (14%), diabetes (11%), cancer (6%), metabolic health (24%), anthropometric indices (28%), fitness (7%), psychological health (8%), cognition (6%), bone health (2%), musculoskeletal problems (2%), sleep (1%), pain (1%), asthma (1%), sciatica (1%), and fatigue (1%). There was no correlation between the number of citations and the number of years since publication (r = 0.082; p = 0.419) i.e., the number of citations was not affected by the year of publication of the paper.

**Table 1:** List of top 100 highly cited sedentary behaviour papers

Rank	Authors	Title	Journal	Article type	Stage of the behavioural epidemiology framework	Citation number	Citation density
1	Matthews et al. (2008)	Amount of time spent in sedentary behaviors in the United States, 2003–2004	American Journal of Epidemiology	Original article (Observational, cross-sectional)	3	1779	127.07
2	Biswas et al. (2015)	Sedentary time and its association with risk for disease incidence, mortality, and hospitalization in adults: a systematic review and meta-analysis	Annals of Internal Medicine	Review (Systematic reviews/meta-analyses)	1	1531	218.71
3	Owen et al. (2010)	Too much sitting: the population health science of sedentary behavior	Exercise and Sport Sciences Reviews	Review (Narrative reviews)	1	1459	121.58
4	Ekelund et al. (2016)	Does physical activity attenuate, or even eliminate, the detrimental association of sitting time with mortality? A harmonised meta-analysis of data from more than 1 million men and women	Lancet	Review (Systematic reviews/meta-analyses)	1	1326	221.00
5	Tremblay et al. (2017)	Sedentary Behavior Research Network (SBRN)—Terminology Consensus Project process and outcome	International Journal of Behavioral Nutrition and Physical Activity	Discussion paper	2	1310	262.00
6	Barnes et al. (2012)	Letter to the editor: standardized use of the terms "sedentary" and "sedentary behaviours"	Applied Physiology, Nutrition, and Metabolism	Discussion paper	2	1279	127.90
7	Robinson TN (1999)	Reducing children's television viewing to prevent obesity: a randomized controlled trial	Journal of the American Medical Association	Original article (Interventional studies)	1 & 5	1280	55.65
8	Tremblay et al. (2011)	Systematic review of sedentary behaviour and health indicators in school-aged children and youth	International Journal of Behavioral Nutrition and Physical Activity	Review (Systematic reviews/meta-analyses)	1 & 3	1228	111.64
9	Hu et al. (2003)	Television watching and other sedentary behaviors in relation to risk of obesity and type 2 diabetes mellitus in women	Journal of the American Medical Association	Original article (Observational, longitudinal)	1	1195	62.89
10	Katzmarzyk et al. (2009)	Sitting time and mortality from all causes, cardiovascular disease, and cancer	Medicine and Science in Sports and Exercise	Original article (Observational, longitudinal)	1	1097	84.38
11	Hamilton et al. (2007)	Role of low energy expenditure and sitting in obesity, metabolic syndrome, type 2 diabetes, and cardiovascular disease	Diabetes	Review (Narrative reviews)	1	1097	73.13
12	Wilmot et al. (2012)	Sedentary time in adults and the association with diabetes, cardiovascular disease and death: systematic review and meta-analysis	Diabetologia	Review (Systematic reviews/meta-analyses)	1	1082	108.20
13	Thorp et al. (2011)	Sedentary behaviors and subsequent health outcomes in adults a systematic review of longitudinal studies, 1996–2011	American Journal of Preventive Medicine	Review (Systematic reviews/meta-analyses)	1	1027	93.36
14	Healy et al. (2008)	Breaks in sedentary time: beneficial associations with metabolic risk	Diabetes Care	Original article (Observational, cross-sectional)	1	1017	72.64
15	Healy et al. (2011)	Sedentary time and cardio-metabolic biomarkers in US adults: NHANES 2003–06	European Heart Journal	Original article (Observational, cross-sectional)	1	951	86.45
16	Dietz et al. (1985)	Do we fatten our children at the television set? Obesity and television viewing in children and adolescents	Pediatrics	Original article (Observational, longitudinal)	1	928	25.08
17	Tremblay et al. (2010)	Physiological and health implications of a sedentary lifestyle	Applied Physiology, Nutrition, and Metabolism	Review (Narrative reviews)	1	855	71.25
18	Pate et al. (2008)	The evolving definition of "sedentary"	Exercise and Sport Sciences Reviews	Discussion paper	2	790	56.43
19	Dunstan et al. (2012)	Breaking up prolonged sitting reduces postprandial glucose and insulin responses	Diabetes Care	Original article (Interventional studies)	1	782	78.20
20	Gortmaker et al. (1996)	Television viewing as a cause of increasing obesity among children in the United States, 1986–1990	Archives of Pediatrics and Adolescent Medicine (now JAMA Pediatrics)	Original article (Observational, longitudinal)	1	771	29.65
21	Carson et al. (2016)	Systematic review of sedentary behaviour and health indicators in school-aged children and youth: an update	Applied Physiology, Nutrition, and Metabolism	Review (Systematic reviews/meta-analyses)	1	653	108.83
22	Grontved et al. (2011)	Television viewing and risk of type 2 diabetes, cardiovascular disease, and all-cause mortality: a meta-analysis	Journal of the American Medical Association	Review (Systematic reviews/meta-analyses)	1	595	54.09
23	van der Ploeg et al. (2012)	Sitting time and all-cause mortality risk in 222 497 Australian adults	Archives of Internal Medicine (now JAMA Internal Medicine)	Original article (Observational, longitudinal)	1	588	58.80
24	Kozey-Keadle et al. (2011)	Validation of wearable monitors for assessing sedentary behavior	Medicine and Science in Sports and Exercise	Original article (Validation papers)	2	578	52.55
25	Dunstan et al. (2010)	Television viewing time and mortality: the Australian Diabetes, Obesity and Lifestyle Study (AusDiab)	Circulation	Original article (Observational, longitudinal)	1	561	46.75
26	Hancox et al. (2004)	Association between child and adolescent television viewing and adult health: a longitudinal birth cohort study	Lancet	Original article (Observational, longitudinal)	1	552	30.67
27	Owen et al. (2011)	Adults' sedentary behavior determinants and interventions	American Journal of Preventive Medicine	Review (Narrative reviews)	4 & 5	537	48.82
28	Hamilton et al. (2008)	Too little exercise and too much sitting: inactivity physiology and the need for new recommendations on sedentary behavior	Current Cardiovascular Risk Reports	Discussion paper	1 & 6	534	38.14
29	Machado de Rezende et al. (2014)	Sedentary behavior and health outcomes: an overview of systematic reviews	PLOS One	Review (Systematic reviews/meta-analyses)	1	522	65.25
30	Dennison et al. (2002)	Television viewing and television in bedroom associated with overweight risk among low-income preschool children	Pediatrics	Original article (Observational, cross-sectional)	1	515	25.75
31	Matthews et al. (2012)	Amount of time spent in sedentary behaviors and cause-specific mortality in US adults	American Journal Of Clinical Nutrition	Original article (Observational, longitudinal)	1	483	48.30
32	Patterson et al. (2018)	Sedentary behaviour and risk of all-cause, cardiovascular and cancer mortality, and incident type 2 diabetes: a systematic review and dose response meta-analysis	European Journal of Epidemiology	Review (Systematic reviews/meta-analyses)	1	475	118.75
33	Owen et al. (2010)	Sedentary behavior: emerging evidence for a new health risk	Mayo Clinic Proceedings	Review (Narrative reviews)	1	472	39.33
34	Proper et al. (2011)	Sedentary behaviors and health outcomes among adults: a systematic review of prospective studies	American Journal of Preventive Medicine	Review (Systematic reviews/meta-analyses)	1	449	40.82
35	Biddle et al. (2010)	Tracking of sedentary behaviours of young people: a systematic review	Preventive Medicine	Review (Systematic reviews/meta-analyses)	3	423	35.25
36	Patel et al. (2010)	Leisure time spent sitting in relation to total mortality in a prospective cohort of US adults	American Journal of Epidemiology	Original article (Observational, longitudinal)	1	406	33.83
37	Healy et al. (2011)	Measurement of adults' sedentary time in population-based studies	American Journal of Preventive Medicine	Review (Narrative reviews)	2 & 3	406	36.91
38	Rey-Lopez et al. (2008)	Sedentary behaviour and obesity development in children and adolescents	Nutrition, Metabolism and Cardiovascular Diseases	Review (Systematic reviews/meta-analyses)	1	404	28.86
39	Bauman et al. (2011)	The descriptive epidemiology of sitting. A 20-country comparison using the International Physical Activity Questionnaire (IPAQ)	American Journal of Preventive Medicine	Original article (Observational, cross-sectional)	3	398	36.18
40	Pearson et al. (2011)	Sedentary behavior and dietary intake in children, adolescents, and adults. A systematic review	American Journal of Preventive Medicine	Review (Systematic reviews/meta-analyses)	4	384	34.91
41	Epstein et al. (2008)	A randomized trial of the effects of reducing television viewing and computer use on body mass index in young children	Archives of Pediatrics and Adolescent Medicine (now JAMA Pediatrics)	Original article (Interventional studies)	1 & 5	382	27.29
42	Manson et al. (2004)	The escalating pandemics of obesity and sedentary lifestyle—A call to action for clinicians	Archives of Internal Medicine (now JAMA Internal Medicine)	Discussion paper	1	366	20.33
43	Warren et al. (2010)	Sedentary behaviors increase risk of cardiovascular disease mortality in men	Medicine and Science in Sports and Exercise	Original article (Observational, longitudinal)	1	357	29.75
44	Epstein et al. (2000)	Decreasing sedentary behaviors in treating pediatric obesity	Archives of Pediatrics and Adolescent Medicine (now JAMA Pediatrics)	Original article (Interventional studies)	1 & 5	357	16.23
45	Dunstan et al. (2012)	Too much sitting–a health hazard	Diabetes Research and Clinical Practice	Review (Narrative reviews)	1	354	35.40
46	Young et al. (2016)	Sedentary behavior and cardiovascular morbidity and mortality: A science advisory from the American Heart Association	Circulation	Review (Narrative reviews)	1	348	58.00
47	van Uffelen et al. (2010)	Occupational sitting and health risks: a systematic review	American Journal of Preventive Medicine	Review (Systematic reviews/meta-analyses)	1	348	29.00
48	Rhodes et al. (2012)	Adult sedentary behavior: a systematic review	American Journal of Preventive Medicine	Review (Systematic reviews/meta-analyses)	3	345	34.50
49	Tremblay et al. (2011)	Canadian guidelines for sedentary behavior to the intention of children and youth	Applied Physiology, Nutrition, and Metabolism	Policy paper	6	345	31.36
50	Bankoski et al. (2011)	Sedentary activity associated with metabolic syndrome independent of physical activity	Diabetes Care	Original article (Observational, cross-sectional)	1	336	30.55
51	Stiglic et al. (2019)	Effects of screentime on the health and well-being of children and adolescents: a systematic review of reviews	BMJ Open	Review (Systematic reviews/meta-analyses)	1	332	110.67
52	Edwardson et al. (2012)	Association of sedentary behaviour with metabolic syndrome: a meta-analysis	PLOS One	Review (Systematic reviews/meta-analyses)	1	334	33.40
53	Atkin et al. (2012)	Methods of measurement in epidemiology: sedentary behaviour	International Journal of Epidemiology	Review (Narrative reviews)	2	332	33.20
54	Ford et al. (2012)	Sedentary behaviour and cardiovascular disease: a review of prospective studies	International Journal of Epidemiology	Review (Systematic reviews/meta-analyses)	1	326	32.60
55	Lis et al. (2007)	Association between sitting and occupational LBP	European Spine Journal	Review (Systematic reviews/meta-analyses)	1	320	21.33
56	Robinson TN (2001)	Television viewing and childhood obesity	Pediatric Clinics of North America	Review (Narrative reviews)	1	319	15.19
57	Healy et al. (2008)	Television time and continuous metabolic risk in physically active adults	Medicine and Science in Sports and Exercise	Original article (Observational, cross-sectional)	1	313	22.36
58	Varo et al. (2003)	Distribution and determinants of sedentary lifestyles in the European Union	International Journal of Epidemiology	Original article (Observational, cross-sectional)	3 & 4	309	16.26
59	Harvey et al. (2015)	How sedentary are older people? A systematic review of the amount of sedentary behavior	Journal of Aging and Physical Activity	Review (Systematic reviews/meta-analyses)	3	292	41.71
60	Teychenne et al. (2011)	Sedentary behavior and depression among adults: a review	International Journal of Behavioral Medicine	Review (Systematic reviews/meta-analyses)	1	293	26.64
61	Zimmerman et al. (2005)	Children's television viewing and cognitive outcomes: a longitudinal analysis of national data	Archives of Pediatrics and Adolescent Medicine (now JAMA Pediatrics)	Original article (Observational, longitudinal)	1	290	17.06
62	Owen et al. (2009)	Too much sitting: a novel and important predictor of chronic disease risk?	British Journal of Sports Medicine	Review (Narrative reviews)	1	286	22.00
63	Lakka et al. (2003)	Sedentary lifestyle, poor cardiorespiratory fitness, and the metabolic syndrome	Medicine and Science in Sports and Exercise	Original article (Observational, cross-sectional)	1	286	15.05
64	Peddie et al. (2013)	Breaking prolonged sitting reduces postprandial glycemia in healthy, normal-weight adults: a randomized crossover trial	American Journal of Clinical Nutrition	Original article (Interventional studies)	1 & 5	276	30.67
65	Certain et al. (2002)	Prevalence, correlates, and trajectory of television viewing among infants and toddlers	Pediatrics	Original article (Observational, longitudinal)	3 & 4	273	13.65
66	Parry et al. (2013)	The contribution of office work to sedentary behaviour associated risk	BMC Public Health	Original article (Observational, cross-sectional)	1	271	30.11
67	Proctor et al. (2003)	Television viewing and change in body fat from preschool to early adolescence: The Framingham Children's Study	International Journal of Obesity	Original article (Observational, longitudinal)	1	271	14.26
68	Alkhajah et al. (2012)	Sit-stand workstations: a pilot intervention to reduce office sitting time	American Journal of Preventive Medicine	Original article (Interventional studies)	5	268	26.80
69	Stamatakis et al. (2011)	Screen-based entertainment time, all-cause mortality, and cardiovascular events: population-based study with ongoing mortality and hospital events follow-up	Journal of the American College of Cardiology	Original article (Observational, longitudinal)	1	268	24.36
70	Diaz et al. (2017)	Patterns of sedentary behavior and mortality in U.S. middle-aged and older adults: a national cohort study	Annals of Internal Medicine	Original article (Observational, longitudinal)	1	263	52.60
71	Rosenberg et al. (2010)	Reliability and validity of the Sedentary Behavior Questionnaire (SBQ) for adults	Journal of Physical Activity and Health	Original article (Validation papers)	2	259	21.58
72	Marshall et al. (2006)	A descriptive epidemiology of screen-based media use in youth: a review and critique	Journal of Adolescence	Review (Systematic reviews/meta-analyses)	3	260	16.25
73	Pate et al. (2011)	Sedentary behaviour in youth	British Journal of Sports Medicine	Review (Systematic reviews/meta-analyses)	3	257	23.36
74	Lauricella et al. (2015)	Young children's screen time: The complex role of parent and child factors	Journal of Applied Developmental Psychology	Original article (Observational, cross-sectional)	4	254	36.29
75	Zhai et al. (2015)	Sedentary behaviour and the risk of depression: a meta-analysis	British Journal of Sports Medicine	Review (Systematic reviews/meta-analyses)	1	251	35.86
76	Buckley et al. (2015)	The sedentary office: an expert statement on the growing case for change towards better health and productivity	British Journal of Sports Medicine	Review (Narrative reviews)	5 & 6	249	35.57
77	Gardner et al. (2016)	How to reduce sitting time? A review of behaviour change strategies used in sedentary behaviour reduction interventions among adults	Health Psychology Review	Review (Systematic reviews/meta-analyses)	5	248	41.33
78	Marshall et al. (2010)	Measuring total and domain-specific sitting: a study of reliability and validity	Medicine and Science in Sports and Exercise	Original article (Validation papers)	2	248	20.67
79	Lynch (2010)	Sedentary behavior and cancer: a systematic review of the literature and proposed biological mechanisms	Cancer Epidemiology, Biomarkers & Prevention	Review (Systematic reviews/meta-analyses)	1	245	20.42
80	Hoare et al. (2016)	The associations between sedentary behaviour and mental health among adolescents: a systematic review	International Journal of Behavioral Nutrition and Physical Activity	Review (Systematic reviews/meta-analyses)	1	243	40.50
81	Healy et al. (2013)	Reducing sitting time in office workers: short-term efficacy of a multicomponent intervention	Preventive Medicine	Original article (Interventional studies)	5	237	26.33
82	O'Donoghue et al. (2016)	A systematic review of correlates of sedentary behaviour in adults aged 18–65 years: a socio-ecological approach	BMC Public Health	Review (Systematic reviews/meta-analyses)	4	231	38.50
83	Prince et al. (2014)	A comparison of the effectiveness of physical activity and sedentary behaviour interventions in reducing sedentary time in adults: a systematic review and meta-analysis of controlled trials	Obesity Reviews	Review (Systematic reviews/meta-analyses)	5	231	28.88
84	Chau et al. (2013)	Daily sitting time and all-cause mortality: a meta-analysis	PLOS One	Review (Systematic reviews/meta-analyses)	1	231	25.67
85	Rosenberg et al. (2008)	Assessment of sedentary behavior with the International Physical Activity Questionnaire	Journal of Physical Activity and Health	Original article (Validation papers)	2	230	16.43
86	Bailey et al. (2015)	Breaking up prolonged sitting with light-intensity walking improves postprandial glycemia, but breaking up sitting with standing does not	Journal of Science and Medicine in Sport	Original article (Interventional studies)	1 & 5	227	32.43
87	Anderson et al. (1985)	Estimates of young children's time with television: a methodological comparison of parent reports with time-lapse video home observation	Child Development	Original article (Validation papers)	2	227	6.14
88	Salmon et al. (2000)	The association between television viewing and overweight among Australian adults participating in varying levels of leisure-time physical activity	International Journal of Obesity	Original article (Observational, cross-sectional)	1	222	10.09
89	Zimmerman et al. (2007)	Television and DVD/video viewing in children younger than 2 years	Archives of Pediatrics and Adolescent Medicine (now JAMA Pediatrics)	Original article (Observational, cross-sectional)	3	222	14.80
90	Lanningham-Foster et al. (2006)	Energy expenditure of sedentary screen time compared with active screen time for children	Pediatrics	Original article (Observational, cross-sectional)	2	221	13.81
91	LeBlanc et al. (2012)	Systematic review of sedentary behaviour and health indicators in the early years (aged 0–4 years)	Applied Physiology, Nutrition, and Metabolism	Review (Systematic reviews/meta-analyses)	1	214	21.40
92	Clark et al. (2009)	Validity and reliability of measures of television viewing time and other non-occupational sedentary behaviour of adults: a review	Obesity Reviews	Review (Systematic reviews/meta-analyses)	2	214	16.46
93	Neuhaus et al. (2014)	Reducing occupational sedentary time: a systematic review and meta-analysis of evidence on activity-permissive workstations	Obesity Reviews	Review (Systematic reviews/meta-analyses)	5	213	26.63
94	Klesges et al. (1993)	Effects of television on metabolic rate: potential implications for childhood obesity	Pediatrics	Original article (Observational, cross-sectional)	1	213	7.34
95	Thorp et al. (2010)	Deleterious associations of sitting time and television viewing time with cardiometabolic risk biomarkers: Australian Diabetes, Obesity and Lifestyle (AusDiab) study 2004–2005	Diabetes Care	Original article (Observational, cross-sectional)	1	210	17.50
96	Harvey et al. (2013)	Prevalence of sedentary behavior in older adults: a systematic review	International Journal of Environmental Research and Public Health	Review (Systematic reviews/meta-analyses)	3	208	23.11
97	Wijndaele et al. (2011)	Television viewing time independently predicts all-cause and cardiovascular mortality: the EPIC Norfolk Study	International Journal of Epidemiology	Original article (Observational, longitudinal)	1	206	18.73
98	Koster et al. (2012)	Association of sedentary time with mortality independent of moderate to vigorous physical activity	PLOS One	Original article (Observational, longitudinal)	1	205	20.50
99	Gorely et al. (2004)	Couch kids: correlates of television viewing among youth	International Journal of Behavioral Medicine	Review (Systematic reviews/meta-analyses)	4	205	11.39
100	Tudor-Locke et al. (2013)	A step-defined sedentary lifestyle index: < 5000 steps/day	Applied Physiology, Nutrition, and Metabolism	Review (Narrative reviews)	2	202	22.44

The most cited paper “*Amount of time spent in sedentary behaviors in the United States, 2003–2004*” was published by Matthews et al. in 2008 in American Journal of Epidemiology and received 1,779 citations (citation density: 127.07). The paper with the highest citation density (i.e., 262) was “*Sedentary Behavior Research Network (SBRN)—Terminology Consensus Project process and outcome*” which was published by Tremblay et al. in 2017 and received 1,310 citations. This was followed by “*Does physical activity attenuate, or even eliminate, the detrimental association of sitting time with mortality? A harmonised meta-analysis of data from more than 1 million men and women*” and “*Sedentary time and its association with risk for disease incidence, mortality, and hospitalization in adults: a systematic review and meta-analysis*” with a citation density of 221 and 218.7, respectively.

As reported in Table 2, of all included publications, 48 reviews received 22,856 citations (citations/paper = 476.2), followed by 46 original research articles with 21,582 citations (citations/paper = 469.2), 5 discussion papers with 4,279 citations (citations/paper = 855.8), and 1 policy paper/guideline with 345 citations (Additional file 1). The highest citation per paper rate was observed for narrative reviews (6,916 citations; 532 citations/paper), followed by observational studies (16,231 citations; 491.8 citations per paper) and interventional studies (3,809 citations; 476.1 citations per paper). The highest citation density, however, was observed for systematic reviews/meta-analyses (15,940 citations; 1939.3 citation density). With respect to behavioural epidemiology framework classification, most papers (n = 64) reported on the relationship of sedentary behaviour with health outcomes and received 34,598 citations (citations/paper = 540.6). Sixty papers were authored through single-country (national) collaboration and 40 through multi-country (international) collaboration.

**Table 2:** Types of papers in the list of top 100 highly cited sedentary behaviour papers

Type of paper	Papers	Number of citations	Citation range (min—max)	CPP	SCC	MCC
1. Original article	46	21,582	205–1779	469.2	34	12
Observational (cross-sectional)	16	7517	210–1779	469.8	10	6
Observational (longitudinal)	17	8714	205–1195	512.6	13	4
Interventional studies	8	3809	227–1280	476.1	7	1
Validation studies	5	1542	227–578	308.4	4	1
2. Review articles	48	22,856	202–1531	476.2	24	24
Narrative reviews	13	6916	202–1459	532	4	9
Systematic reviews/meta-analyses	35	15,940	205–1531	455.4	20	15
3. Discussion papers	5	4279	366–1310	855.8	2	3
4. Policy papers	1	345	345–345	345	0	1
Behavioural epidemiology framework stage						
Relationship of sedentary behaviour with health outcomes	64	34,598	205–1531	540.6	40	24
Measurement of sedentary behaviour	13	6296	202–1310	484.3	7	6
Prevalence and epidemiology of sedentary behaviour	12	5172	208–1779	431	8	4
Determinants and correlates of sedentary behaviour	7	2193	205–537	313.3	2	5
Interventions and programs to influence sedentary behaviour	7	1983	213–537	283.3	4	3
Policy and practice in sedentary behaviour and public health	2	594	249–345	297	0	2

*CPP* Citations per paper, *SCC* Single-country (national) collaboration, *MCC* Multi-country (international) collaboration

### Journal of publication

Overall, 44 journals published the 100 most cited sedentary behaviour papers, with 50% papers published in the 10 most prolific journals (Table 3). The journal American Journal of Preventive Medicine published 9 (9%) papers and received 4,162 citations (462.4 citations/paper). The highest cited paper was published in the International Journal of Epidemiology which was ranked as the eighth most prolific journal. Eight of the top 10 journals were Q1 journals in their respective category, and were owned by or affiliated with a specialty-specific organization or society. The journal distribution shows that the papers were published in public health, sport sciences, paediatrics, health-related speciality (i.e., endocrinology and metabolism, nutrition and dietetics, cardiac and cardiovascular systems, psychology, and orthopaedics), general or internal medicine, and multidisciplinary sciences journals. The impact factors of the top 10 journals publishing the 100 most cited sedentary behaviour papers ranged from 3.06 to 157.38, with Journal Citation Indicator values ranging from 0.81 to 10.46. Overall, the impact factor of journals publishing the 100 most cited papers ranged from 2.11 to 202.73 (median = 6.64), with 67 papers published in Q1 journals, 29 in Q2, 3 in Q3, and 1 in Q4 journals in the journal citation reports. We found a significant correlation between the journal impact factor and the number of citations (r = 0.27; p = 0.008).

**Table 3:** Top 10 Journals that published 100 highly cited sedentary behaviour papers

Rank	Journal	Papers	Number of citations	CPP	Affiliation	JIF_2021_	JCI_2021_	Quartile	Category
1	American Journal of Preventive Medicine	9	4162	462.44	American College of Preventive Medicine and the Association for Prevention Teaching and Research	6.604	1.6	Q1	Public, Environmental, and Occupational Health
2	Medicine and Science in Sports and Exercise	6	3548	591.33	American College of Sports Medicine	3.061	0.81	Q2	Sport Science
3	Applied Physiology, Nutrition, And Metabolism	6	2879	479.83	Canadian Society for Exercise Physiology, the Canadian Nutrition Society, and Exercise & Sports Science Australia	6.289	1.83	Q1	Sport Science
4	Archives of Pediatrics and Adolescent Medicine (now JAMA Pediatrics)	5	2022	404.40	–	26.8	5.77	Q1	Pediatrics
5	Pediatrics	5	2150	430.00	American Academy of Pediatrics	9.703	3.17	Q1	Pediatrics
6	British Journal of Sports Medicine	4	1043	260.75	Several	18.479	3.69	Q1	Sport Science
7	Diabetes Care	4	2345	586.25	The American Diabetes Association	17.155	3.55	Q1	Endocrinology & Metabolism
8	International Journal of Epidemiology	4	1173	293.25	International Epidemiological Association	9.685	2.47	Q1	Public, Environmental, and Occupational Health
9	PLOS One	4	1292	323.00	–	3.752	0.88	Q2	Multidisciplinary Sciences
10	Journal of the American Medical Association	3	3070	1023.33	American Medical Association	157.375	10.46	Q1	Medicine, General & Internal

*CPP* Citations per paper, *JIF*_*2021*_ Journal impact factor for 2021, *JCI*_*2021*_ Journal citation indicator for 2021

### Contributing authors

Overall, top 10 most prolific authors held the first (lead) author role in 21 and last (senior) author role in 31 papers (Table 4). In 58 papers, top 10 highly prolific authors were listed as co-authors (i.e., not as first or last author). Eight of the top 10 authors had a current affiliation from Australia, 2 from Canada, and 1 from the USA. Owen N published 23 (23%) papers, with 13,813 citations and 600.6 citations per paper. While Owen had the highest number (n = 10) of last (senior) author papers, Healy GN, who was ranked as third most prolific author, published the most (n = 5) first author papers. Saunders TJ had the highest citations per paper (855.4) with 4,277 citations on only 5 papers.

**Table 4:** Top 10 Authors that contributed to publishing 100 highly cited sedentary behaviour papers

Rank	Author	Papers	Number of citations	CPP	Co-author	First author	Last author	Current affiliation
1	Owen N	23	13,813	600.57	9	4	10	Swinburne University of Technology, Australia
2	Dunstan DW	17	9209	541.71	8	3	6	Deakin University, Australia
3	Healy GN	17	9194	540.82	10	5	2	University of Queensland, Australia
4	Salmon J	9	3944	438.22	7	1	1	Deakin University, Australia
5	Biddle SJH	8	3268	408.50	0	1	7	University of Southern Queensland, Australia
6	Matthews CE	8	5986	748.25	4	2	2	National Cancer Institute, USA
7	Tremblay MS	7	5142	734.57	1	4	2	Children’s Hospital of Eastern Ontario, Canada
8	Bauman A	6	3168	528.00	4	1	1	University of Sydney, Australia
9	Saunders TJ	5	4277	855.40	5	0	0	University of Prince Edward Island, Canada
10	Shaw JE	5	2883	576.60	5	0	0	Baker Heart and Diabetes Institute, Australia
10	Zimmet PZ	5	2883	576.60	5	0	0	Monash University, Australia

*CPP* Citations per paper

**Fig. 3:**
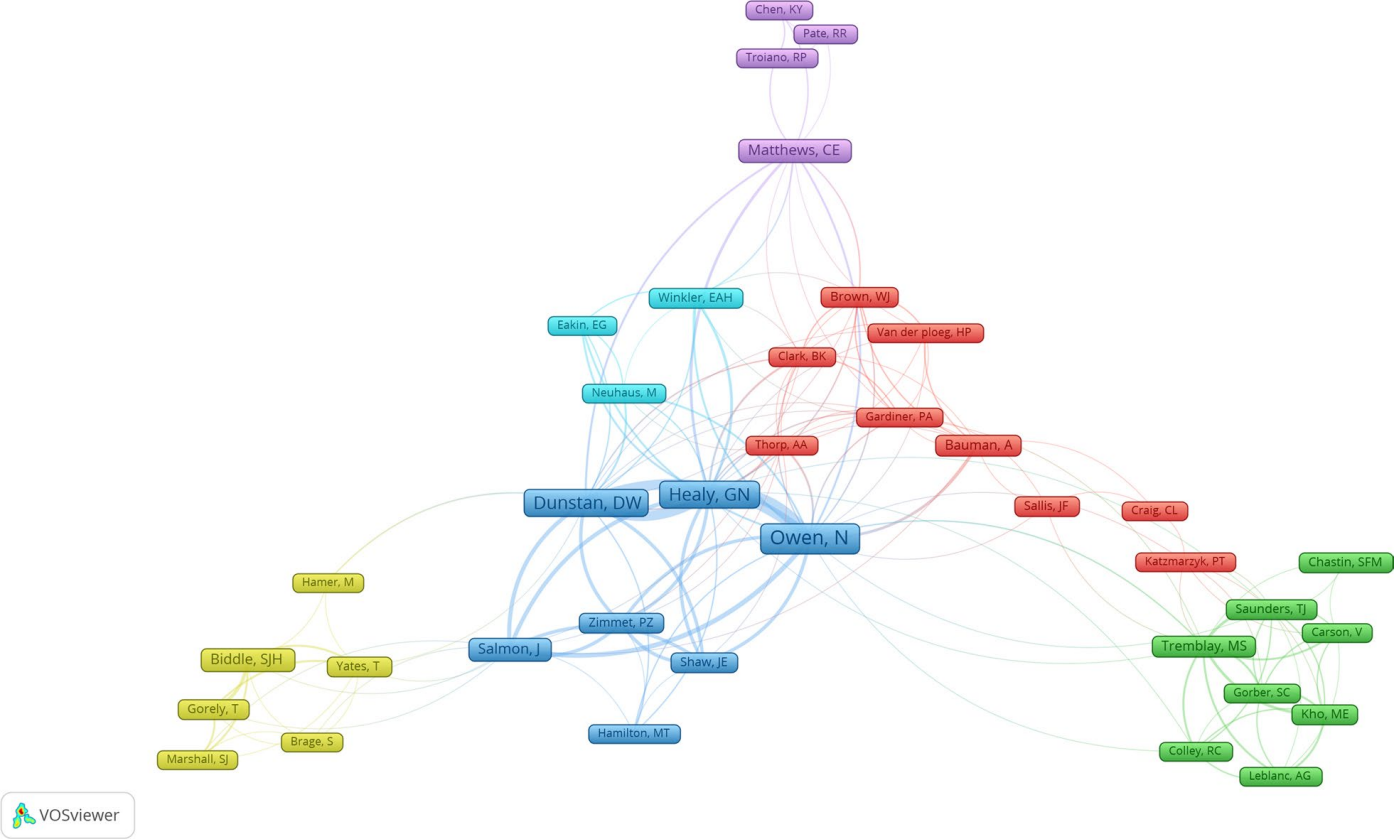
Co-authorship network visualization map for authors (with a minimum of 3 papers) of 100 highly cited sedentary behaviour papers. Interactive map may be accessed here: https://tinyurl.com/2g84c2sw

Co-authorship network analysis produced a map for authors with a minimum of 3 papers and showed 37 authors in 6 clusters (Fig. 3). Each cluster was shown with a specific colour showing co-authorship in published papers. For example, Owen N, Healy DN, Dunstan DW, Salmon J, Zimmet PZ, Shaw JE, and Hamilton MT appeared in the same (blue) cluster. The visualization map showed that Owen N had 21 links on 23 papers (i.e., collaboration with 21 authors in the map) and a link strength of 82 (i.e., they appeared in different sequences for a total of 82 times as co-authors). Healy GN had 19 links and a link strength of 75 for 17 papers, whereas Dunstan DW had 18 links and a link strength of 69 for 17 papers.

### Contributing countries and institutions

Based on the institutional address of the author, individuals from 17 countries contributed to the 100 highly cited sedentary behaviour papers. Among these, the United States had 55 papers, with 29,811 citations (542.02 citations/paper). Other countries with over 10 publications included Australia (n = 37), UK (n = 23), and Canada (n = 15). Although the USA received 29,811 citations on 55, the highest citations per paper was 837 for Norway with 1,674 on just 2 papers (Table 5). Four of the top 10 contributing countries were from Europe, 3 from Asia–Pacific, 2 from North America, and 1 from South America.

**Table 5:** Top 10 Countries that contributed to publishing 100 highly cited sedentary behaviour papers

Rank	Country	Papers	Number of citations	CPP
1	USA	55	29,811	542.02
2	Australia	37	18,175	491.22
3	UK	23	10,375	451.09
4	Canada	15	10,470	698.00
5	Netherlands	6	2762	460.33
6	New Zealand	3	2107	702.33
7	China	3	2050	683.33
8	Norway	2	1674	837.00
9	Brazil	2	997	498.50
10	Denmark	2	800	400.00

*CPP* Citations per paper

Four of the top 10 institutions contributing to the 100 most cited papers were from Australia, four from the United States, two from Canada and one the United Kingdom. University of Queensland (Australia) was the top institution with 24 publications and 13,862 citations (577.6 citations/paper) contributing to the 100 most cited sedentary behaviour papers. Harvard T.H. Chan School of Public Health had the highest citations per paper (i.e., 863.5) with 5,181 citations on only 6 papers (Table 6).

**Table 6:** Top 10 Institutions that contributed to publishing 100 highly cited sedentary behaviour papers

Rank	Affiliation	Country	Papers	Number of citations	CPP
1	University of Queensland	Australia	24	13,862	577.58
2	Baker Heart and Diabetes Institute	Australia	15	8928	595.20
3	Deakin University	Australia	14	7231	516.50
4	Loughborough University	UK	10	4777	477.70
5	University of Ottawa	Canada	9	6652	739.11
6	National Institutes of Health (NIH)	USA	9	6322	702.44
6	National Cancer Institute (NCI)	USA	9	6322	702.44
7	Children's Hospital of Eastern Ontario	Canada	8	6421	802.63
8	University of Sydney	Australia	8	3609	451.13
9	Harvard T.H. Chan School of Public Health	USA	6	5181	863.50
10	Pennington Biomedical Research Center	USA	6	3377	562.83

*CPP* Citations per paper

### Keywords

**Fig. 4:**
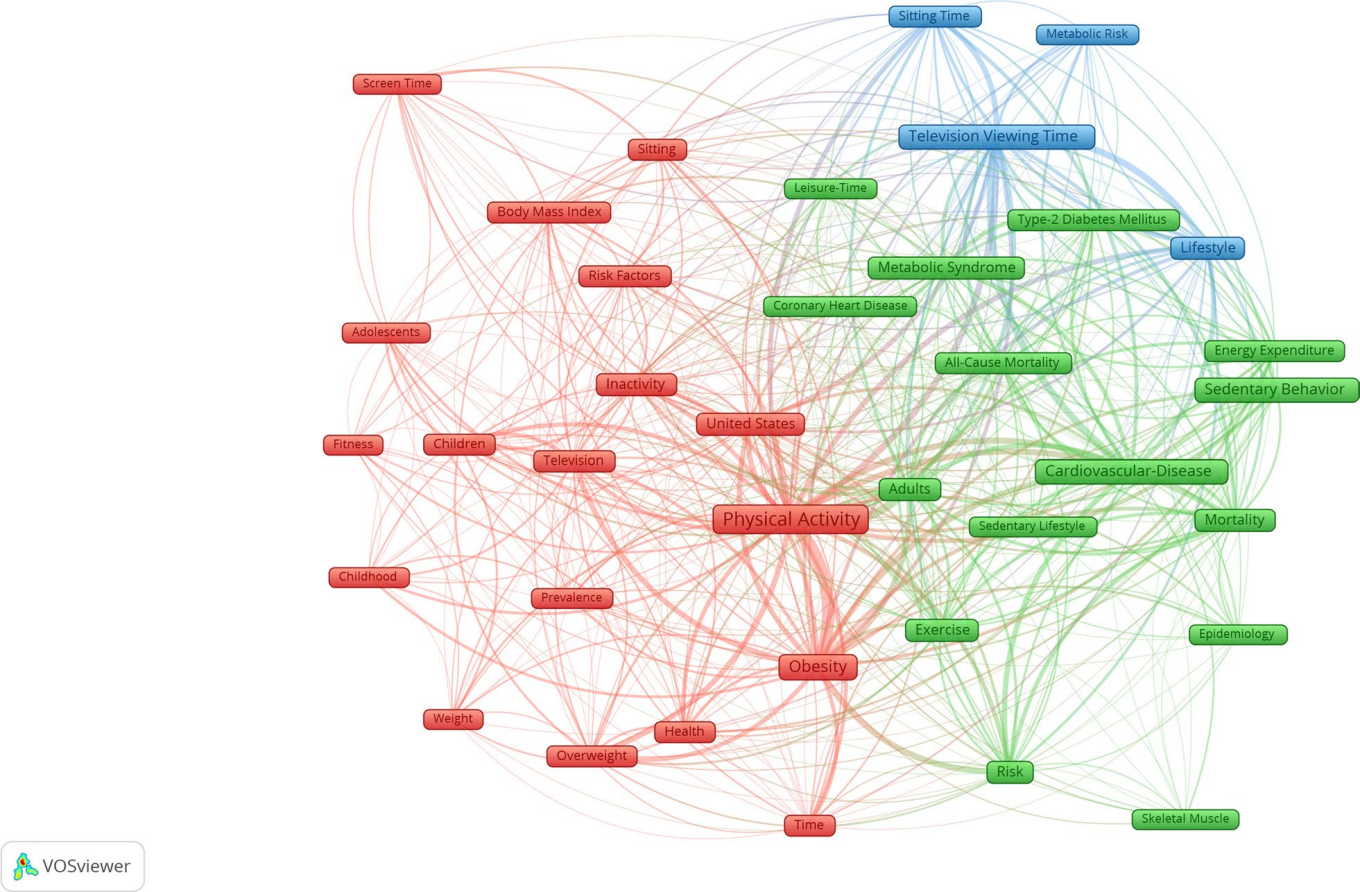
Visualization map for keywords (with a minimum occurrence of 6 times) of 100 highly cited sedentary behaviour papers. Interactive map may be accessed here: https://tinyurl.com/2oxrgyek

The map for keywords with occurrences of ≥ 6 times produced 3 clusters of 37 keywords in total (Fig. 4). The most common keywords included: physical-activity, obesity, cardiovascular-disease, television viewing time, adults, United-States, life-style, metabolic syndrome, risk, and exercise. Sedentary behaviour specific keywords included television, television viewing time, sitting, sitting time, sedentary behaviour (including variations such as sedentary behaviors or sedentary behaviour), screen time, and sedentary lifestyle. Overall, almost all keywords in the map were on the relationship of sedentary behaviour with health, sedentary behaviour prevalence and epidemiology, measurement of sedentary and behaviour.

## Discussion

In this study, we identified 100 most cited sedentary behaviour papers and their bibliometric characteristics, including the most prolific authors, journals, countries, and institutions. The citation analysis demonstrates the way the field has grown over time. Overall, 100 most cited sedentary behaviour papers received 49,062 citations, with most citations received by observational studies, and most studies on the relationship of sedentary behaviour with health outcomes. Most studies were through single country (national) collaboration and were in the early stages of the Behavioural Epidemiology framework. Finally, most papers were published by authors affiliated with institutions from high-income countries.

The 100 most cited sedentary behaviour papers were cited between 202 and 1,779 times. This is lower when compared with citations for publications on physical activity (range = 297 – 8,068), despite including a wider range of studies (i.e., 500) [39]. This is most likely due to the physical activity field being much larger, hence more papers will receive more citations. Furthermore, 100 top cited papers in some fields such as hypertension (range = 582 – 7,248), diabetes (range = 964 – 17,779), and acute kidney injury (range = 215 – 1,971) also received more citations [34, 45, 46]. Nevertheless, the citations to the 100 most cited sedentary behaviour papers were more than clinical orthopaedic sports medicine (range = 229 – 1,629) [47]. This is not surprising because the citation pattern varies across different fields based on the scope of the field, the number of researchers and journals or several other factors (e.g., journal publishing model and indexing, primary language and geographic origin of the authors) [1, 39]. Nevertheless, almost no highly cited sedentary behaviour papers were published before 2000 and most were published after 2010, this illustrates that this is a very young field of research that has expanded very rapidly in a short time. Forty eight percent of included studies were systematic reviews, this is comparable to our study on most cited physical activity papers which included 39% reviews [39]. The higher proportion of reviews as highly cited studies is explained by systematic reviews being at the highest hierarchical level of evidence, thus they are more likely to get cited compared to original studies.

The majority (77%) of the most cited sedentary behaviour papers were published after 2007. Of note, prior to 2008 sedentary behaviour was used interchangeably for physical inactivity. However, these fields started to segregate with some discussion papers showing that sedentary behaviour is distinct health behaviour – brining more clarity to physical inactivity vs sedentary distinction [19, 25]. This distinction was made further clear during 2010s with some publications showing that physical activity (or inactivity) and sedentary behaviour are interrelated but separate health behaviours [26–28]. As such, it may be argued that the landmark paper on physical activity that was published by Morris et al. in 1953 rather looked at sedentary behaviour, instead of physical activity, and mortality at work [17]. Noticeably, we did not find a correlation between the number of citations and the number of years since publication. Given that over half (n = 52) of the included papers used some form of open access, this finding is unexpected. The rapid rise in the field, particularly during 2010s, bringing more clarity to physical inactivity vs sedentary distinction might have caused more recent papers becoming highly cited in a way that is proportionate to older ones. While this tends to vary across different fields, future research is needed to explain this finding in more detail.

Strikingly, only one policy paper made it into the 100 most cited sedentary behaviour papers [48]. This may be because papers combining physical activity and sedentary behaviour and papers examining 24-h movement behaviours were not included in this bibliometric analysis. Moreover, the majority (n = 64) of highly cited papers belong to the first stage of the Behavioural Epidemiology Framework (i.e., relationships with health), which suggests that there has been sufficient research output indicating the detrimental effects and associations of sedentary behaviour. Most health outcomes were related to physical health (anthropometric indices, metabolic health, and mortality). However, there have been less high impact studies examining outcomes of interventions in randomised controlled trials demonstrating the benefit of reducing sedentary behaviour, which are needed to ultimately guide policy and public health implementation. Likewise, there have been less high impact studies on determinants and correlates of sedentary behaviour which may help identify targets and contexts for these intervention trials.

Almost all the most cited sedentary behaviour papers were published by authors from high-income countries, with either two or three publications from middle- or low-income countries (e.g., Brazil, China, and Kenya). This is, however, not surprising because such trend was also observed in physical activity research and other medical fields [39, 45, 49]. Higher research output from high-income countries may be due to a higher number of researchers, higher research funding, and stronger research collaborations [45, 49]. This was further supported by an observation that most prolific authors tend to collaborate with authors from other high-income countries. For example, Owen N had the strongest research links with researchers from other high-income countries, including Tremblay MS, Matthews CE, Van der ploeg HP and others (Fig. 3). Countries such as the USA, UK, Australia and Canada are at the top in terms of most cited sedentary behaviour papers because such countries have better economic ranking, infrastructure, and research support [39, 49]. In addition, it has been argued that researchers from high-income countries tend to cite researchers from the same country or similar income group which may be attributed to the way these researchers have collaborative networks within the field [39, 49, 50]. However, relative to the size of its population and number of active researchers, the proliferation of contributions from Australia (ranked second in this study) is remarkable. This may be attributed to the fact that several of the initial leaders of the sedentary behaviour field (i.e., those that were among the first to make a conceptual distinction between physical inactivity and sedentary behaviour and associated health outcomes) were based in Australia. On the other hand, a lack of sedentary behaviour research in low-income countries may be attributed to a lack of researchers or research focusing on this field. In addition, a higher proportion of work in low- and middle-income countries is characterized as physical labour compared to high-income countries where there is a greater service industry resulting in more sedentary office-based work [49]. As such, sedentary behaviour research is less likely to have been prioritised in low- and middle-income countries.

This is the first bibliometric study presenting the 100 most cited sedentary behaviour papers without restriction to language and time of publication. However, some of the limitations of the study should be acknowledged. The search of all indexes of Web of Science was comprehensive and covered Medline, but there were inaccuracies in data compared to Web of Science Core Collection. For example, there were duplicate author and institution names in bibliometric parameters. More information for the differences between Web of Science Core Collection and Web of Science All Databases (indexes) may be found elsewhere [51].F urther, we only included studies with main focus on sedentary behaviour, so studies with a focus on physical activity or sleep combined with sedentary behaviour were not included in the analysis. Although we included studies where physical activity was considered one of the ‘other’ variables (e.g., covariates, mediators, moderators) in papers focusing on sedentary behaviour (e.g., *Does physical activity attenuate, or even eliminate, the detrimental association of sitting time with mortality?*), we acknowledge that this approach might have excluded some seminal papers that combine sedentary behaviour with physical activity or sleep. For example, papers by Tremblay et al. [52] Bull et al. [15] and Tremblay et al. [53] were not included in the analysis. Furthermore, the bibliometric parameters obtained from Web of Science database may differ from other databases used for bibliometric analyses (e.g., Scopus) because they differ in journals and other sources (e.g., books) indexed. Although there would be a large overlap for most cited sedentary behaviour papers in Web of Science compared to Scopus, the list of the 100 most cited sedentary behaviour papers may not be generalizable to other databases. Although we extracted open access information from the Web of Science database, it is likely that some journals have changed their publishing model over time. We also extracted information about the study population, number of authors, multi-country collaborations, and specific health outcomes. However, examining additional variables (i.e., study design, setting) was not related to the main objective and research question of this study. As this study was focused on sedentary behaviour only, we were unable to examine 24-h movement behaviours (which is now also informing guidelines). Therefore, we suggest that future research should consider examining most cited 24-h movement behaviour research papers.

## Conclusions

Systematic reviews and discussion papers have had the most impact on sedentary behaviour research. Papers on the relationship of sedentary behaviour with health outcomes are cited more often, and research on measurement, correlates and interventions was poorly represented. Papers published after 2007 were predominant in the list of 100 most cited sedentary behaviour papers. The most cited sedentary behaviour studies were predominantly focused on adult population, and on physical health outcomes (e.g., anthropometric indices, metabolic health, and mortality). The most influential institutions and authors are from high-income countries, such as the United States, Australia, the United Kingdom, Canada, and some European countries. Future bibliometric studies should consider adding additional variables such as the publishing model of the journal, population groups, health outcomes, correlates or determinants in addition to other bibliometric parameters.

## Supplementary Information

**Additional file 1. **1 search strategy.

**Additional file 2. **List of 100 highly cited papers stratified by type.

## Data Availability

The data used during the current study are available from the corresponding author on reasonable request.
