# Supplementary material for: Vigorously cited: a bibliometric analysis of the 100 most cited sedentary behaviour articles
Source: J Act Sedentary Sleep Behav. 2023 Jul 1;2:13. doi: 10.1186/s44167-023-00022-8 (PMC11960230; doi:10.1186/s44167-023-00022-8)
Supplement: Supplementary file 1 — Additional file 1. 1 search strategy. [file 44167_2023_22_MOESM1_ESM.docx]

Additional file 1

Search strategy

Step 1: 32,986

TI=(accelerometer OR "activity behav*" OR "cell phone use" OR driv* OR "energy balance" OR "energy expenditure" OR "everyday activities" OR "intenet use" OR "media use" OR "mobile phone use" OR "movement and play behav*" OR "screen time" OR "screen-based entertainment time" OR "screen-time" OR sedentariness OR sedentary OR "sedentary behavior" OR "sedentary behaviour" OR "sedentary lifestyle" OR "sedentary time" OR sitting OR "sitting time" OR "social media use" OR "television and video game use" OR "television exposure" OR "television time" OR "television viewing" OR "television watching" OR "television-viewing" OR "time use" OR "TV viewing" OR "use of television") AND AB=("reclining time" OR SB OR sedentariness OR sedentary OR "sedentary behav*" OR "sedentary lifestyle" OR "sedentary time" OR sitting OR "stationary behav*") OR KP=("reclining time" OR SB OR sedentariness OR sedentary OR "sedentary behav*" OR "sedentary lifestyle" OR "sedentary time" OR sitting OR "stationary behav*")

Step 2: 104,552

TI=(sedentariness OR "sedentary behav*" OR sitting OR "reclining time" OR "stationary behav*" OR "sedentary time" OR "cell phone use" OR "mobile use") OR AB=(sedentariness OR "sedentary behav*" OR sitting OR "reclining time" OR "stationary behav*" OR "sedentary time" OR "cell phone use" OR "mobile use") OR KP=(sedentariness OR "sedentary behav*" OR sitting OR "reclining time" OR "stationary behav*" OR "sedentary time" OR "cell phone use" OR "mobile use")

Step 3: 20,499

TI=("watching TV" OR "television watching" OR "TV watching" OR "TV viewing" OR television OR "video watching" OR "watching video" OR "internet use" OR gaming OR "video games" OR "video?game" OR "social media" OR "screen time" OR "small screen" OR "electronic game playing" OR "media time" OR "media use" OR "electronic media" OR "smartphone? use" OR "mobile use" OR "mobile phone use" OR "cell phone" OR "app use" OR "PC use" OR "PC time" OR "computer use" OR "computer time" OR "tablet use" OR "tablet time") AND AB=(sedentariness OR "sedentary behav*" OR sitting OR "reclining time" OR "stationary behav*" OR "sedentary time" OR "computer use" OR "media screen activities" OR "media use" OR "mobile phone use" OR "mobile phone exposure" OR "social media use" OR "screen time" OR "screen-time" OR "television viewing" OR "television-viewing" OR "TV viewing" OR "television watching" OR "TV watching" OR "TV use" OR "use of digital media" OR "viewing TV" OR "viewing television" OR "watching television") OR KP=(sedentariness OR "sedentary behav*" OR sitting OR "reclining time" OR "stationary behav*" OR "sedentary time" OR "media screen activities" OR "media use" OR "mobile phone use" OR "mobile phone exposure" OR "social media use" OR "screen time" OR "screen-time" OR "television viewing" OR "television-viewing" OR "television watching" OR "TV watching" OR "TV viewing" OR "TV use" OR "use of digital media" OR "viewing TV" OR "viewing television" OR "watching television")

Step 4: 123,090

#1 OR #2 OR #3
