# Supplementary material for: Vigorously cited: a bibliometric analysis of the 100 most cited sedentary behaviour articles
Source: J Act Sedentary Sleep Behav. 2023 Jul 1;2:13. doi: 10.1186/s44167-023-00022-8 (PMC11960230; doi:10.1186/s44167-023-00022-8)
Supplement: Supplementary file 2 — Additional file 2. List of 100 highly cited papers stratified by type. [file 44167_2023_22_MOESM2_ESM.docx]

Additional file 2: Table List of 100 top cited sedentary behaviour articles, stratified by article type

| Rank | Authors | Title | Journal | Article type | Stage of the behavioural epidemiology framework | Citation number | Citation density |
| --- | --- | --- | --- | --- | --- | --- | --- |
| Discussion papers | | | | | | | |
| 5 | Tremblay et al (2017) | Sedentary Behavior Research Network (SBRN) - Terminology Consensus Project process and outcome | International Journal of Behavioral Nutrition and Physical Activity | Discussion paper | 2 | 1310 | 262 |
| 6 | Barnes et al (2012) | Letter to the editor: standardized use of the terms "sedentary" and "sedentary behaviours" | Applied Physiology, Nutrition, and Metabolism | Discussion paper | 2 | 1279 | 127.9 |
| 18 | Pate et al (2008) | The evolving definition of "sedentary" | Exercise and Sport Sciences Reviews | Discussion paper | 2 | 790 | 56.43 |
| 28 | Hamilton et al (2008) | Too little exercise and too much sitting: inactivity physiology and the need for new recommendations on sedentary behavior | Current Cardiovascular Risk Reports | Discussion paper | 1 & 6 | 534 | 38.14 |
| 42 | Manson et al (2004) | The escalating pandemics of obesity and sedentary lifestyle - A call to action for clinicians | Archives of Internal Medicine (now JAMA Internal Medicine) | Discussion paper | 1 | 366 | 20.33 |
| Original article (Interventional studies) | | | | | | | |
| 7 | Robinson TN (1999) | Reducing children's television viewing to prevent obesity: a randomized controlled trial | Journal of the American Medical Association | Original article (Interventional studies) | 1 & 5 | 1280 | 55.65 |
| 19 | Dunstan et al (2012) | Breaking up prolonged sitting reduces postprandial glucose and insulin responses | Diabetes Care | Original article (Interventional studies) | 1 | 782 | 78.2 |
| 41 | Epstein et al (2008) | A randomized trial of the effects of reducing television viewing and computer use on body mass index in young children | Archives of Pediatrics and Adolescent Medicine (now JAMA Pediatrics) | Original article (Interventional studies) | 1 & 5 | 382 | 27.29 |
| 44 | Epstein et al (2000) | Decreasing sedentary behaviors in treating pediatric obesity | Archives of Pediatrics and Adolescent Medicine (now JAMA Pediatrics) | Original article (Interventional studies) | 1 & 5 | 357 | 16.23 |
| 64 | Peddie et al (2013) | Breaking prolonged sitting reduces postprandial glycemia in healthy, normal-weight adults: a randomized crossover trial | American Journal of Clinical Nutrition | Original article (Interventional studies) | 1 & 5 | 276 | 30.67 |
| 68 | Alkhajah et al (2012) | Sit-stand workstations: a pilot intervention to reduce office sitting time | American Journal of Preventive Medicine | Original article (Interventional studies) | 5 | 268 | 26.8 |
| 81 | Healy et al (2013) | Reducing sitting time in office workers: short-term efficacy of a multicomponent intervention | Preventive Medicine | Original article (Interventional studies) | 5 | 237 | 26.33 |
| 86 | Bailey et al (2015) | Breaking up prolonged sitting with light-intensity walking improves postprandial glycemia, but breaking up sitting with standing does not | Journal of Science and Medicine in Sport | Original article (Interventional studies) | 1 & 5 | 227 | 32.43 |
| Original article (Observational, cross-sectional) | | | | | | | |
| 1 | Matthews et al (2008) | Amount of time spent in sedentary behaviors in the United States, 2003-2004 | American Journal of Epidemiology | Original article (Observational, cross-sectional) | 3 | 1779 | 127.07 |
| 14 | Healy et al (2008) | Breaks in sedentary time: beneficial associations with metabolic risk | Diabetes Care | Original article (Observational, cross-sectional) | 1 | 1017 | 72.64 |
| 15 | Healy et al (2011) | Sedentary time and cardio-metabolic biomarkers in US adults: NHANES 2003-06 | European Heart Journal | Original article (Observational, cross-sectional) | 1 | 951 | 86.45 |
| 30 | Dennison et al (2002) | Television viewing and television in bedroom associated with overweight risk among low-income preschool children | Pediatrics | Original article (Observational, cross-sectional) | 1 | 515 | 25.75 |
| 39 | Bauman et al (2011) | The descriptive epidemiology of sitting. A 20-country comparison using the International Physical Activity Questionnaire (IPAQ) | American Journal of Preventive Medicine | Original article (Observational, cross-sectional) | 3 | 398 | 36.18 |
| 50 | Bankoski et al (2011) | Sedentary activity associated with metabolic syndrome independent of physical activity | Diabetes Care | Original article (Observational, cross-sectional) | 1 | 336 | 30.55 |
| 57 | Healy et al (2008) | Television time and continuous metabolic risk in physically active adults | Medicine and Science in Sports and Exercise | Original article (Observational, cross-sectional) | 1 | 313 | 22.36 |
| 58 | Varo et al (2003) | Distribution and determinants of sedentary lifestyles in the European Union | International Journal of Epidemiology | Original article (Observational, cross-sectional) | 3 & 4 | 309 | 16.26 |
| 63 | Lakka et al (2003) | Sedentary lifestyle, poor cardiorespiratory fitness, and the metabolic syndrome | Medicine and Science in Sports and Exercise | Original article (Observational, cross-sectional) | 1 | 286 | 15.05 |
| 66 | Parry et al (2013) | The contribution of office work to sedentary behaviour associated risk | BMC Public Health | Original article (Observational, cross-sectional) | 1 | 271 | 30.11 |
| 74 | Lauricella et al (2015) | Young children's screen time: The complex role of parent and child factors | Journal of Applied Developmental Psychology | Original article (Observational, cross-sectional) | 4 | 254 | 36.29 |
| 88 | Salmon et al (2000) | The association between television viewing and overweight among Australian adults participating in varying levels of leisure-time physical activity | International Journal of Obesity | Original article (Observational, cross-sectional) | 1 | 222 | 10.09 |
| 89 | Zimmerman et al (2007) | Television and DVD/video viewing in children younger than 2 years | Archives of Pediatrics and Adolescent Medicine (now JAMA Pediatrics) | Original article (Observational, cross-sectional) | 3 | 222 | 14.8 |
| 90 | Lanningham-Foster et al (2006) | Energy expenditure of sedentary screen time compared with active screen time for children | Pediatrics | Original article (Observational, cross-sectional) | 2 | 221 | 13.81 |
| 94 | Klesges et al (1993) | Effects of television on metabolic rate: potential implications for childhood obesity | Pediatrics | Original article (Observational, cross-sectional) | 1 | 213 | 7.34 |
| 95 | Thorp et al (2010) | Deleterious associations of sitting time and television viewing time with cardiometabolic risk biomarkers: Australian Diabetes, Obesity and Lifestyle (AusDiab) study 2004-2005 | Diabetes Care | Original article (Observational, cross-sectional) | 1 | 210 | 17.5 |
| Original article (Observational, longitudinal) | | | | | | | |
| 9 | Hu et al (2003) | Television watching and other sedentary behaviors in relation to risk of obesity and type 2 diabetes mellitus in women | Journal of the American Medical Association | Original article (Observational, longitudinal) | 1 | 1195 | 62.89 |
| 10 | Katzmarzyk et al (2009) | Sitting time and mortality from all causes, cardiovascular disease, and cancer | Medicine and Science in Sports and Exercise | Original article (Observational, longitudinal) | 1 | 1097 | 84.38 |
| 16 | Dietz et al (1985) | Do we fatten our children at the television set? Obesity and television viewing in children and adolescents | Pediatrics | Original article (Observational, longitudinal) | 1 | 928 | 25.08 |
| 20 | Gortmaker et al (1996) | Television viewing as a cause of increasing obesity among children in the United States, 1986-1990 | Archives of Pediatrics and Adolescent Medicine (now JAMA Pediatrics) | Original article (Observational, longitudinal) | 1 | 771 | 29.65 |
| 23 | van der Ploeg et al (2012) | Sitting time and all-cause mortality risk in 222 497 Australian adults | Archives of Internal Medicine (now JAMA Internal Medicine) | Original article (Observational, longitudinal) | 1 | 588 | 58.8 |
| 25 | Dunstan et al (2010) | Television viewing time and mortality: the Australian Diabetes, Obesity and Lifestyle Study (AusDiab) | Circulation | Original article (Observational, longitudinal) | 1 | 561 | 46.75 |
| 26 | Hancox et al (2004) | Association between child and adolescent television viewing and adult health: a longitudinal birth cohort study | Lancet | Original article (Observational, longitudinal) | 1 | 552 | 30.67 |
| 31 | Matthews et al (2012) | Amount of time spent in sedentary behaviors and cause-specific mortality in US adults | American Journal Of Clinical Nutrition | Original article (Observational, longitudinal) | 1 | 483 | 48.3 |
| 36 | Patel et al (2010) | Leisure time spent sitting in relation to total mortality in a prospective cohort of US adults | American Journal of Epidemiology | Original article (Observational, longitudinal) | 1 | 406 | 33.83 |
| 43 | Warren et al (2010) | Sedentary behaviors increase risk of cardiovascular disease mortality in men | Medicine and Science in Sports and Exercise | Original article (Observational, longitudinal) | 1 | 357 | 29.75 |
| 61 | Zimmerman et al (2005) | Children's television viewing and cognitive outcomes: a longitudinal analysis of national data | Archives of Pediatrics and Adolescent Medicine (now JAMA Pediatrics) | Original article (Observational, longitudinal) | 1 | 290 | 17.06 |
| 65 | Certain et al (2002) | Prevalence, correlates, and trajectory of television viewing among infants and toddlers | Pediatrics | Original article (Observational, longitudinal) | 3 & 4 | 273 | 13.65 |
| 67 | Proctor et al (2003) | Television viewing and change in body fat from preschool to early adolescence: The Framingham Children's Study | International Journal of Obesity | Original article (Observational, longitudinal) | 1 | 271 | 14.26 |
| 69 | Stamatakis et al (2011) | Screen-based entertainment time, all-cause mortality, and cardiovascular events: population-based study with ongoing mortality and hospital events follow-up | Journal of the American College of Cardiology | Original article (Observational, longitudinal) | 1 | 268 | 24.36 |
| 70 | Diaz et al (2017) | Patterns of sedentary behavior and mortality in U.S. middle-aged and older adults: a national cohort study | Annals of Internal Medicine | Original article (Observational, longitudinal) | 1 | 263 | 52.6 |
| 97 | Wijndaele et al (2011) | Television viewing time independently predicts all-cause and cardiovascular mortality: the EPIC Norfolk Study | International Journal of Epidemiology | Original article (Observational, longitudinal) | 1 | 206 | 18.73 |
| 98 | Koster et al (2012) | Association of sedentary time with mortality independent of moderate to vigorous physical activity | PLOS One | Original article (Observational, longitudinal) | 1 | 205 | 20.5 |
| Original article (Validation papers) | | | | | | | |
| 24 | Kozey-Keadle et al (2011) | Validation of wearable monitors for assessing sedentary behavior | Medicine and Science in Sports and Exercise | Original article (Validation papers) | 2 | 578 | 52.55 |
| 71 | Rosenberg et al (2010) | Reliability and validity of the Sedentary Behavior Questionnaire (SBQ) for adults | Journal of Physical Activity and Health | Original article (Validation papers) | 2 | 259 | 21.58 |
| 78 | Marshall et al (2010) | Measuring total and domain-specific sitting: a study of reliability and validity | Medicine and Science in Sports and Exercise | Original article (Validation papers) | 2 | 248 | 20.67 |
| 85 | Rosenberg et al (2008) | Assessment of sedentary behavior with the International Physical Activity Questionnaire | Journal of Physical Activity and Health | Original article (Validation papers) | 2 | 230 | 16.43 |
| 87 | Anderson et al (1985) | Estimates of young children's time with television: a methodological comparison of parent reports with time-lapse video home observation | Child Development | Original article (Validation papers) | 2 | 227 | 6.14 |
| Policy papers | | | | | | | |
| 49 | Tremblay et al (2011) | Canadian guidelines for sedentary behavior to the intention of children and youth | Applied Physiology, Nutrition, and Metabolism | Policy papers | 6 | 345 | 31.36 |
| Review (Narrative reviews) | | | | | | | |
| 3 | Owen et al (2010) | Too much sitting: the population health science of sedentary behavior | Exercise and Sport Sciences Reviews | Review (Narrative reviews) | 1 | 1459 | 121.58 |
| 11 | Hamilton et al (2007) | Role of low energy expenditure and sitting in obesity, metabolic syndrome, type 2 diabetes, and cardiovascular disease | Diabetes | Review (Narrative reviews) | 1 | 1097 | 73.13 |
| 17 | Tremblay et al (2010) | Physiological and health implications of a sedentary lifestyle | Applied Physiology, Nutrition, and Metabolism | Review (Narrative reviews) | 1 | 855 | 71.25 |
| 27 | Owen et al (2011) | Adults' sedentary behavior determinants and interventions | American Journal of Preventive Medicine | Review (Narrative reviews) | 4 & 5 | 537 | 48.82 |
| 33 | Owen et al (2010) | Sedentary behavior: emerging evidence for a new health risk | Mayo Clinic Proceedings | Review (Narrative reviews) | 1 | 472 | 39.33 |
| 37 | Healy et al (2011) | Measurement of adults' sedentary time in population-based studies | American Journal of Preventive Medicine | Review (Narrative reviews) | 2 & 3 | 406 | 36.91 |
| 45 | Dunstan et al (2012) | Too much sitting--a health hazard | Diabetes Research and Clinical Practice | Review (Narrative reviews) | 1 | 354 | 35.4 |
| 46 | Young et al (2016) | Sedentary behavior and cardiovascular morbidity and mortality: A science advisory from the American Heart Association | Circulation | Review (Narrative reviews) | 1 | 348 | 58 |
| 53 | Atkin et al (2012) | Methods of measurement in epidemiology: sedentary behaviour | International Journal of Epidemiology | Review (Narrative reviews) | 2 | 332 | 33.2 |
| 56 | Robinson TN (2001) | Television viewing and childhood obesity | Pediatric Clinics of North America | Review (Narrative reviews) | 1 | 319 | 15.19 |
| 62 | Owen et al (2009) | Too much sitting: a novel and important predictor of chronic disease risk? | British Journal of Sports Medicine | Review (Narrative reviews) | 1 | 286 | 22 |
| 76 | Buckley et al (2015) | The sedentary office: an expert statement on the growing case for change towards better health and productivity | British Journal of Sports Medicine | Review (Narrative reviews) | 5 & 6 | 249 | 35.57 |
| 100 | Tudor-Locke et al (2013) | A step-defined sedentary lifestyle index: < 5000 steps/day | Applied Physiology, Nutrition, and Metabolism | Review (Narrative reviews) | 2 | 202 | 22.44 |
| Review (Systematic reviews/meta-analyses) | | | | | | | |
| 2 | Biswas et al (2015) | Sedentary time and its association with risk for disease incidence, mortality, and hospitalization in adults: a systematic review and meta-analysis | Annals of Internal Medicine | Review (Systematic reviews/meta-analyses) | 1 | 1531 | 218.71 |
| 4 | Ekelund et al (2016) | Does physical activity attenuate, or even eliminate, the detrimental association of sitting time with mortality? A harmonised meta-analysis of data from more than 1 million men and women | Lancet | Review (Systematic reviews/meta-analyses) | 1 | 1326 | 221 |
| 8 | Tremblay et al (2011) | Systematic review of sedentary behaviour and health indicators in school-aged children and youth | International Journal of Behavioral Nutrition and Physical Activity | Review (Systematic reviews/meta-analyses) | 1 & 3 | 1228 | 111.64 |
| 12 | Wilmot et al (2012) | Sedentary time in adults and the association with diabetes, cardiovascular disease and death: systematic review and meta-analysis | Diabetologia | Review (Systematic reviews/meta-analyses) | 1 | 1082 | 108.2 |
| 13 | Thorp et al (2011) | Sedentary behaviors and subsequent health outcomes in adults a systematic review of longitudinal studies, 1996-2011 | American Journal of Preventive Medicine | Review (Systematic reviews/meta-analyses) | 1 | 1027 | 93.36 |
| 21 | Carson et al (2016) | Systematic review of sedentary behaviour and health indicators in school-aged children and youth: an update | Applied Physiology, Nutrition, and Metabolism | Review (Systematic reviews/meta-analyses) | 1 | 653 | 108.83 |
| 22 | Grontved et al (2011) | Television viewing and risk of type 2 diabetes, cardiovascular disease, and all-cause mortality: a meta-analysis | Journal of the American Medical Association | Review (Systematic reviews/meta-analyses) | 1 | 595 | 54.09 |
| 29 | Machado de Rezende et al (2014) | Sedentary behavior and health outcomes: an overview of systematic reviews | PLOS One | Review (Systematic reviews/meta-analyses) | 1 | 522 | 65.25 |
| 32 | Patterson et al (2018) | Sedentary behaviour and risk of all-cause, cardiovascular and cancer mortality, and incident type 2 diabetes: a systematic review and dose response meta-analysis | European Journal of Epidemiology | Review (Systematic reviews/meta-analyses) | 1 | 475 | 118.75 |
| 34 | Proper et al (2011) | Sedentary behaviors and health outcomes among adults: a systematic review of prospective studies | American Journal of Preventive Medicine | Review (Systematic reviews/meta-analyses) | 1 | 449 | 40.82 |
| 35 | Biddle et al (2010) | Tracking of sedentary behaviours of young people: a systematic review | Preventive Medicine | Review (Systematic reviews/meta-analyses) | 3 | 423 | 35.25 |
| 38 | Rey-Lopez et al (2008) | Sedentary behaviour and obesity development in children and adolescents | Nutrition, Metabolism and Cardiovascular Diseases | Review (Systematic reviews/meta-analyses) | 1 | 404 | 28.86 |
| 40 | Pearson et al (2011) | Sedentary behavior and dietary intake in children, adolescents, and adults. A systematic review | American Journal of Preventive Medicine | Review (Systematic reviews/meta-analyses) | 4 | 384 | 34.91 |
| 47 | van Uffelen et al (2010) | Occupational sitting and health risks: a systematic review | American Journal of Preventive Medicine | Review (Systematic reviews/meta-analyses) | 1 | 348 | 29 |
| 48 | Rhodes et al (2012) | Adult sedentary behavior: a systematic review | American Journal of Preventive Medicine | Review (Systematic reviews/meta-analyses) | 3 | 345 | 34.5 |
| 51 | Stiglic et al (2019) | Effects of screentime on the health and well-being of children and adolescents: a systematic review of reviews | BMJ Open | Review (Systematic reviews/meta-analyses) | 1 | 332 | 110.67 |
| 52 | Edwardson et al (2012) | Association of sedentary behaviour with metabolic syndrome: a meta-analysis | PLOS One | Review (Systematic reviews/meta-analyses) | 1 | 334 | 33.4 |
| 54 | Ford et al (2012) | Sedentary behaviour and cardiovascular disease: a review of prospective studies | International Journal of Epidemiology | Review (Systematic reviews/meta-analyses) | 1 | 326 | 32.6 |
| 55 | Lis et al (2007) | Association between sitting and occupational LBP | European Spine Journal | Review (Systematic reviews/meta-analyses) | 1 | 320 | 21.33 |
| 59 | Harvey et al (2015) | How sedentary are older people? A systematic review of the amount of sedentary behavior | Journal of Aging and Physical Activity | Review (Systematic reviews/meta-analyses) | 3 | 292 | 41.71 |
| 60 | Teychenne et al (2011) | Sedentary behavior and depression among adults: a review | International Journal of Behavioral Medicine | Review (Systematic reviews/meta-analyses) | 1 | 293 | 26.64 |
| 72 | Marshall et al (2006) | A descriptive epidemiology of screen-based media use in youth: a review and critique | Journal of Adolescence | Review (Systematic reviews/meta-analyses) | 3 | 260 | 16.25 |
| 73 | Pate et al (2011) | Sedentary behaviour in youth | British Journal of Sports Medicine | Review (Systematic reviews/meta-analyses) | 3 | 257 | 23.36 |
| 75 | Zhai et al (2015) | Sedentary behaviour and the risk of depression: a meta-analysis | British Journal of Sports Medicine | Review (Systematic reviews/meta-analyses) | 1 | 251 | 35.86 |
| 77 | Gardner et al (2016) | How to reduce sitting time? A review of behaviour change strategies used in sedentary behaviour reduction interventions among adults | Health Psychology Review | Review (Systematic reviews/meta-analyses) | 5 | 248 | 41.33 |
| 79 | Lynch BM (2010) | Sedentary behavior and cancer: a systematic review of the literature and proposed biological mechanisms | Cancer Epidemiology, Biomarkers & Prevention | Review (Systematic reviews/meta-analyses) | 1 | 245 | 20.42 |
| 80 | Hoare et al (2016) | The associations between sedentary behaviour and mental health among adolescents: a systematic review | International Journal of Behavioral Nutrition and Physical Activity | Review (Systematic reviews/meta-analyses) | 1 | 243 | 40.5 |
| 82 | O'Donoghue et al (2016) | A systematic review of correlates of sedentary behaviour in adults aged 18-65 years: a socio-ecological approach | BMC Public Health | Review (Systematic reviews/meta-analyses) | 4 | 231 | 38.5 |
| 83 | Prince et al (2014) | A comparison of the effectiveness of physical activity and sedentary behaviour interventions in reducing sedentary time in adults: a systematic review and meta-analysis of controlled trials | Obesity Reviews | Review (Systematic reviews/meta-analyses) | 5 | 231 | 28.88 |
| 84 | Chau et al (2013) | Daily sitting time and all-cause mortality: a meta-analysis | PLOS One | Review (Systematic reviews/meta-analyses) | 1 | 231 | 25.67 |
| 91 | LeBlanc et al (2012) | Systematic review of sedentary behaviour and health indicators in the early years (aged 0-4 years) | Applied Physiology, Nutrition, and Metabolism | Review (Systematic reviews/meta-analyses) | 1 | 214 | 21.4 |
| 92 | Clark et al (2009) | Validity and reliability of measures of television viewing time and other non-occupational sedentary behaviour of adults: a review | Obesity Reviews | Review (Systematic reviews/meta-analyses) | 2 | 214 | 16.46 |
| 93 | Neuhaus et al (2014) | Reducing occupational sedentary time: a systematic review and meta-analysis of evidence on activity-permissive workstations | Obesity Reviews | Review (Systematic reviews/meta-analyses) | 5 | 213 | 26.63 |
| 96 | Harvey et al (2013) | Prevalence of sedentary behavior in older adults: a systematic review | International Journal of Environmental Research and Public Health | Review (Systematic reviews/meta-analyses) | 3 | 208 | 23.11 |
| 99 | Gorely et al (2004) | Couch kids: correlates of television viewing among youth | International Journal of Behavioral Medicine | Review (Systematic reviews/meta-analyses) | 4 | 205 | 11.39 |
